# PICH deficiency limits the progression of MYC-induced B-cell lymphoma

**DOI:** 10.1038/s41408-024-00979-y

**Published:** 2024-01-23

**Authors:** María Castejón-Griñán, Eliene Albers, Lucía Simón-Carrasco, Paula Aguilera, Mauro Sbroggio, David Pladevall-Morera, Andreas Ingham, Ernest Lim, Alba Guillen-Benitez, Elena Pietrini, Michael Lisby, Ian D. Hickson, Andres J. Lopez-Contreras

**Affiliations:** 1 Centro Andaluz de Biología Molecular y Medicina Regenerativa (CABIMER), Consejo Superior de Investigaciones Científicas (CSIC), Universidad de Sevilla - Universidad Pablo de Olavide, Seville, Spain; 2 Center for Chromosome Stability and Center for Healthy Aging, Department of Cellular and Molecular Medicine, University of Copenhagen, Copenhagen, Denmark; 3 Center for Chromosome Stability, Department of Biology, University of Copenhagen, Copenhagen, Denmark

**Keywords:** Oncogenesis, Cancer models, Cancer therapy

## Abstract

Plk1-interacting checkpoint helicase (PICH) is a DNA translocase involved in resolving ultrafine anaphase DNA bridges and, therefore, is important to safeguard chromosome segregation and stability. PICH is overexpressed in various human cancers, particularly in lymphomas such as Burkitt lymphoma, which is caused by MYC translocations. To investigate the relevance of PICH in cancer development and progression, we have combined novel PICH-deficient mouse models with the Eμ-Myc transgenic mouse model, which recapitulates B-cell lymphoma development. We have observed that PICH deficiency delays the onset of MYC-induced lymphomas in *Pich* heterozygous females. Moreover, using a *Pich* conditional knockout mouse model, we have found that *Pich* deletion in adult mice improves the survival of Eμ-Myc transgenic mice. Notably, we show that *Pich* deletion in healthy adult mice is well tolerated, supporting PICH as a suitable target for anticancer therapies. Finally, we have corroborated these findings in two human Burkitt lymphoma cell lines and we have found that the death of cancer cells was accompanied by chromosomal instability. Based on these findings, we propose PICH as a potential therapeutic target for Burkitt lymphoma and for other cancers where PICH is overexpressed.

## Introduction

Burkitt lymphoma (BL) is the most frequent non-Hodgkin’s malignant lymphoma (NHML) to affect children in Europe, and also accounts for 2% of adult NHML [1]. It is an aggressive tumor that arises in various parts of the lymphatic system and is one of the fastest-growing tumors [2]. Although most patients respond well to standard chemotherapy, some patients, especially adults, with relapsed disease have a poor prognosis [3]. Thus, it is still necessary to identify mechanisms and molecular targets that may provide early prognostic markers and/or critical targets for the development of new drugs that block BL initiation and progression. In this regard, targeting pathways involved in the maintenance of chromosomal stability has emerged in the last few years as a promising cancer research area [4].

Faithful chromosomal segregation is critical for the maintenance of genomic stability in every cell division. Polo-like kinase 1 (Plk1)-interacting checkpoint helicase (PICH), encoded by the gene *ERCC6L*, is required for the resolution of anaphase Ultrafine DNA Bridges (UFBs) and hence for proper chromosome segregation. PICH localizes in the cytosol during interphase but associates with the nuclear DNA after the nuclear envelope breaks down during mitosis [5, 6]. PICH is a DNA translocase with high affinity for DNA under tension [7] and characteristically localizes to UFBs and contributes to their resolution [5, 8]. UFBs are catenated DNA structures derived from unresolved DNA replication intermediates that link the sister chromatids of mitotic chromosomes in anaphase [9, 10]. UFBs can arise from different chromosomal loci which are difficult to replicate: telomeres, centromeres, common fragile sites, and ribosomal DNA [9–11]. Failure in the resolution of UFBs at the end of anaphase may lead to chromosomal instability in the daughter cells [8, 12]. UFBs may also play beneficial roles in the maintenance of chromosome structure in rapidly proliferating cells [9]. Since UFBs are dechromatinized and cannot be stained with DNA dyes like DAPI, they can only be visualized via proteins that are associated with them, such as PICH or the Bloom’s syndrome helicase (BLM) [8, 10]. PICH recruits other UFB-associated factors such as BLM or RIF1 [6, 8] and stimulates the catalytic activity of the TOP2α topoisomerase in order to resolve UFBs [12, 13].

We have recently generated a *Pich* knock-out (KO) mouse model that contributed to understanding PICH function in vivo [14]. PICH is essential for embryonic development since the loss of *Pich* generates chromosomal instability, DNA damage, p53 activation, and apoptosis in *Pich* KO embryos that are incompatible with sustained viability [14]. More recently, it has been proposed that PICH supports embryonic hematopoiesis by suppressing a cGAS-STING-mediated type I interferon production [15].

The contribution of PICH deficiency-induced chromosomal instability to tumorigenesis in vivo is largely unknown. In fact, whether chromosomal instability (CIN) causes tumor development is still a matter of debate [16, 17]. In contrast, our previous findings indicate that PICH is of critical importance for supporting intense bouts of cell proliferation such as those that occur during embryonic development and the proliferation of transformed cells [14]. Recently, several papers support the notion that PICH activity is relevant to sustain the proliferation of certain types of cancerous cells [18–25]. In addition, PICH is overexpressed in several types of solid human cancers [18, 19, 21–28]. High expression of PICH has been linked to worse outcomes and decreased overall survival in cancer patients [14, 18, 19, 21, 23, 24, 27–29]. However, the role of PICH in hematopoietic malignancies and, in particular, in BL has not been explored yet.

In this study, we have investigated the consequences of PICH depletion in adult mice; and the impact of PICH depletion in BL, using mouse models and human BL cells. Our results indicate that PICH is highly expressed in BL. We also show that *Pich* deficiency delays the development of *Myc*-induced B-cell lymphoma. Furthermore, inducible systemic *Pich* deletion in adult Eµ-Myc mice increases their survival without causing toxic effects. PICH-depleted tumors derived from these mice and human BL cells have a significantly elevated frequency of apoptosis, persistent DNA bridges, bi- and poly nucleation, and micronuclei. Thus, we demonstrate that PICH is required for the growth of *Myc*-induced B-cell lymphoma in vivo and for human BL cells in vitro. In view of these results, we propose PICH as a potential novel target for the treatment of human BL.

## Methods

### Analysis of PICH expression from human databases

*PICH* mRNA expression in human cell lines and tissues was obtained from Cancer Cell Line Encyclopedia (CCLE) and the TCGA, respectively. RNA-seq expression data from TCGA (http://cancergenome.nih.gov/) were obtained through GEPIA (http://gepia.cancer-pku.cn/) to compare *PICH* expression levels between tumors and paired normal tissues. PICH mRNA expression in human samples from different subtypes of mature B-cell malignancies were obtained from an MD Anderson Cancer Center [30] study via cBioportal. DepMap portal (https://depmap.org/portal/) was used to analyze *PICH* expression levels across various human cancer cell lines from the Cancer Cell Lines Encyclopedia.

### Mice

The *Pich* conditional KO (PICH cKO) and constitutive *Pich* KO mouse models were generated in our laboratory and have been described previously [14]. The *Pich* cKO strain has a mixed 129S2; C57BL/6 N background. The UBC-Cre-ERT2 (Jax strain 007001) and Eµ-Myc (Jax strain 002728) [31] strains have been described previously. Activation of the inducible UBC-Cre-ERT2 recombinase was achieved by injecting the *Pich* cKO with Tamoxifen (TAM, T5648, Sigma-Aldrich) into the peritoneum once every 24 h for 5 consecutive days followed by one injection per week in the subsequent 2 weeks. All mice were housed under standard conditions at the animal facility of the Department of Experimental Medicine at the University of Copenhagen and the research was monitored by the Institutional Animal Care and Use Committee. All animal experiments were approved by the Danish Animal Ethics Committee (licenses 2016-15-0201-011111 and 2019-15-0201-00344) and performed in compliance with Danish and European regulations.

### Mouse genotyping

Mice were genotyped for *Pich* gene using three primers as described in [14] *Pich*_F1 (5′-CTATGCCTGATCCTCCCCAG-3′), *Pich*_R1 (5′-GCTAACAGACAAAATGGCCCT-3′) and *Pich*_F2 (5′-AAAGCCCAACTACAGTGTGG-3′). A 390-bp WT fragment and a 495-bp *Pich*^Lox^ fragment were amplified using primers *Pich* _F1 and *Pich* _R1. The *Pich* KO allele was amplified by the *Pich* _R1 and *Pich* _F2 primer and generated a 220-bp fragment. Other primers used for genotyping were g*Cre*5 (5′-TGGTTTCCCGCAGAACCTGAAG-3′ and g*Cre*3: 5′-GAGCCTGTTTTGCACGTTCACC-3′) for *Cre* gene amplification, and OIMR0375 (5′-CAGCTGGCGTAATAGCGAAGAG-3′), OIMR0376 (5′- CTGTGACTGGTGAGTACTCAACC-3′), Tshb_Fw (5′- CTA CAT GAG CAG GCA GAC TGG A-3′) and Tshb_Rv (5′-AAT GGA CGG GTG CTT CTA TC-3′) for amplification of the *Myc* gene.

### Immunohistochemistry

Tissues were fixed in 4% neutral buffered formalin (Sigma-Aldrich), embedded in paraffin and processed according to standard procedures. Immunohistochemistry (IHC) of mouse samples was carried out as described previously [14]. The primary antibodies used for mouse samples were PICH (Cell Signaling Technology, 8886, D4G8; 1:50;), γH2AX (MilliporeSigma, 05-636, JBV301; 1:25,000;) and cleaved caspase-3 (Cell Signaling Technology, 9661; 1:750).

The human lymphoma tissue microarray (TMA), was acquired from US Biomax (LM482c). Paraffin-embedded sections were deparaffinized in xylene and rehydrated through graded concentrations of ethanol in water. Following antigen retrieval with citrate buffer (pH 6.0), endogenous peroxidase was blocked using 3% hydrogen peroxide and sections were incubated overnight at 4 °C with anti-PICH (MyBiosource, MBS2519675, 1:100). Sections were then washed and incubated with secondary antibody for 30 min. IHC reaction was developed using 3,30-diaminobenzidine tetrahydrochloride (DAB) (Chromomap DAB, Ventana, Roche; DAB+ Chromogen System, Dako) and nuclei were counterstained with hematoxylin. Images were acquired using a slide scanner (AxioScan Z1, Zeiss).

### Cell culture

Raji and Ramos BL cells were grown in RPMI (Roswell Park Memorial Institute) medium supplemented with 10% of fetal bovine serum (FBS) (Life Technologies) and 1% of penicillin/streptomycin (p/s) (Life Technologies). HEK293T cells were grown in DMEM supplemented with 10% of FBS, 1% of p/s and 1% of l-glutamin. All cells were incubated at 5% CO_2_ and 37 °C in a ThermoQuest incubator (Forma Scientific, Marietta, OH, USA).

### Western blotting

Cells were lysed in RIPA buffer (Tris-HCl 50 mM, pH 7.4, NP-40 1%, Na-deoxycholate 0.25%, NaCl: 150 mM, EDTA 1 mM) supplemented with complete protease inhibitor cocktail tablet (Roche), 5 mM β-glycerophosphate (Sigma), 5 mM sodium fluoride (Sigma) and 1 mM sodium orthovanadate (Sigma). The total amount of protein was determined by DC Protein Assay kit (Bio-Rad) according to supplier’s instructions. Cell lysates containing 30 µg protein were incubated in NuPAGE™ LDS Sample Buffer 4x (Novex) with 10 mM DTT for 15 min at 70 °C. Samples were resolved by SDS-PAGE and Western blotting was carried out as described previously [32]. Antibodies against PICH (Cell Signaling Technology, 8886; 1:1000) and β-actin (Sigma, A5441; 1:1000) were used. Goat anti-rabbit immunoglobulin G (IgG) HRP (Sigma, A6667; 1:10000) and goat anti-mouse IgG HRP (Sigma-Aldrich, A4416; 1:10000) were used as secondary antibodies. Images were acquired on an Amersham™ Imager 600 (GE Healthcare Life Sciences). Measurements for the protein of interest were normalized to the loading control.

### Lentivirus synthesis

HEK293T cells were used for the synthesis of third-generation lentiviruses containing short hairpin RNA (shRNA) targeting human *PICH* (shPICH) or the shRNA control pLKO.1 (shCtrl) (#8453, Addgene) vector. Briefly, 6 × 10^6^ of cells were reverse transfected using lipofectamine 2000 with 10 μg of the plasmid of interest and the plasmids coding for the lentivirus packaging components (6.5 µg pMMDLRRE, 2.5 µg PRSVREV and 3.5 µg PMDGVSVG) (#12251, #12253 and #12259, respectively, Addgene). Forty-eight hours post-transfection, the viruses were filtered (0.25 µm) and collected. shPICH were acquired from Sigma (MISSION® shRNA Bacterial Glycerol Stock, SHCLNG-NM_017669) and their sequences were as follows: shPICH-1 5’-TATTCTGAGCACTAGCTTAAT-3’; shRNA-2 5’- ACAAGATCTCTCCAGTATAAA -3’; shRNA-3 5’- GATCATGCCAACCAATCTTAT -3’.

### Cell Infection

A total of 2 × 10^5^ Raji or Ramos cells were infected with lentivirus containing the vector of interest and 10 µg/ml of polybrene to facilitate the infection and were incubated for 3 h. Then, the cells and lentivirus mix were then seeded into a 12-well plate. Two days after transduction, cells were selected with 2 µg/mL of puromycin for three days. The selected cells were cultured and used as indicated.

### Cell proliferation assay

Cells were seeded in triplicates in 12-well plates at a density of 5 × 10^5^ cells per well. Cells were mixed with trypan blue (1:1 ratio) (Sigma) for staining and manually counted using a hemocytometer at the time points indicated in the figures over 7 days after selection with puromycin. Non-stained live cell numbers were plotted using GraphPad Prism software (GraphPad Software, San Diego, CA, USA, www.graphpad.com). Doubling times (DT) were calculated by non-linear regression using an exponential growth equation.

### Viability assays

Three independent methods were used to evaluate the viability of *PICH* knockdown (KD) cells. For all methods, cells were seeded six days after lentiviral infection and viability was assessed four days later. Three technical replicates were seeded for each condition. For manual counting, cells were seeded and stained with trypan blue as described above. For the 3-(4,5-dimethylthiazol-2-yl)-2,5-diphenyltetrazoliumbromide (MTT) assay, cells were seeded in 96-well plates at a density of 5 × 10^4^ cells per well. Three days later, cells were incubated with MTT solution (Sigma-Aldrich, 1 mg/ml) for four hours. Formazan was solubilized in DMSO and absorbances at 570 nm and at 690 nm (background) were measured using a Varioskan plate reader (Thermo Fisher Scientific). Normalized absorbance values relative to shCtrl cells were plotted using GraphPad Prism. Finally, Hoechst 33342 and To-Pro-3 staining assay was performed to evaluate the number of total and dead cells, respectively. PICH KD cells were seeded in µCLEAR 96-well plates (Grenier Bio-One) at a density of 1.5 × 10^4^ cells per well. Next, cells were incubated with Hoechst 33342 and To-Pro-3 at a final concentration of 5 µg/ml and 1 µM, respectively, for 30 min in a 5% CO_2_ incubator at 37 °C. Images were acquired automatically by a High-Content Screening System (MetaXpress) and analyzed with the MetaXpress High-Content Image Analysis Software (Molecular Devices). The number of living cells was calculated by subtracting the number of dead cells, given by the To-Pro-3 stain, from the total cell number, given by the Hoechst stain, and plotted using GraphPad Prism.

### Immunofluorescence and microscopy

Fifty thousand cells per well were seeded in triplicates in µCLEAR 96-well plates (Grenier Bio-One) and left steady for 30 min. Cells were fixed with 4% formaldehyde (VWR Chemicals, Radnor, PA, USA) for 15 min at room temperature (RT) and permeabilized with 0.5% Triton X-100 (*v/v*) in PBS for 10 min. Then, cells were washed twice with PBS supplemented with 0.05% Tween-20, blocked with 3% BSA (Sigma) for 30 min and then labeled with α-tubulin primary antibody (Sigma, St. Louis, MO, USA, T9026, 1:500). Next, fluorescence-tagged secondary antibody (Alexa Fluor™ Goat Anti-mouse IgG 488 (Invitrogen) was added for 2 h at RT in the dark. DAPI was used for nuclear staining. Images were automatically acquired using a High-Content Screening System (MetaExpress) and analyzed with the MetaXpress software (Molecular Devices). Single plane images corresponding to Z positions of the maximal DAPI signal were acquired. At least nine images were acquired per well.

### Annexin V-FITC kit

Apoptosis was evaluated by flow cytometry analysis using an Annexin V-FITC apoptosis detection kit from Miltenyi Biotec (Bergisch Gladbach, Germany) following the manufacturer’s instructions. Briefly, 1 × 10^6^ cells were resuspended in binding buffer, stained with Annexin V-FITC antibody for 15 min in the dark and washed with binding buffer. Next, cells were stained with propidium iodide (PI) immediately prior to analysis in a FACSCalibur cytometer from BD Biosciences (San Jose, CA, USA). Data were analyzed using BD CellQuest Pro Software.

### Cell cycle analysis

A total of 1 × 10^6^ cells were fixed in ice-cold 70% ethanol in PBS, then pelleted and resuspended in PBS containing 100 µg/mL RNase A and 20 µg/mL PI(Sigma P4170) for 30 min. Cells were captured by a FACSCalibur cytometer from BD Biosciences (San Jose, CA, USA) and analyzed using *ModFit* Software (Verity Software House).

### EdU incorporation

The DNA replication rate was determined by EdU incorporation using Click-iT technology following the manufacturer’s protocol (Life Technologies). Briefly, 5 × 10^5^ cells were seeded in a T25 flask the day before the staining. EdU (Life Technologies A10044) was added to the culture medium at a final concentration of 10 μM for 60 min prior to fixing the cells with 4% formaldehyde (VWR Chemicals, Radnor, PA, USA) and permeabilizing with 0.1% Triton X-100 (*v/v*) in PBS. Next, a dilution of 1 mM ascorbic acid was prepared fresh, and a Click-it reaction mix was performed by mixing PBS, 2 mM CuSO_4_, 2.5 µg/mL Azide 488, and 1 mM ascorbic acid in the given order, which was then added to each cell condition for 60 min (protected from light). Cells were stained with 20 µg/mL PI and treated with 100 µg/mL RNAse for 30 min at room temperature and analyzed by FACSCalibur cytometer. Data were plotted and analyzed using *ModFit* Software (Verity Software House).

### Statistical analysis

Statistical analyses were performed using GraphPad Prism 8 (GraphPad Software, San Diego California, USA, www.graphpad.com). The significance was determined by either an unpaired *t*-test or one-way ANOVA with Tukey post-test. The log-rank test was used to determine *P* values for all Kaplan–Meier survival curve analyses. *P*-values are indicated in each graph and figure legends. In vitro experiments were performed with at least three biological replicates.

## Results

### PICH is overexpressed in BL

**Fig. 1:**
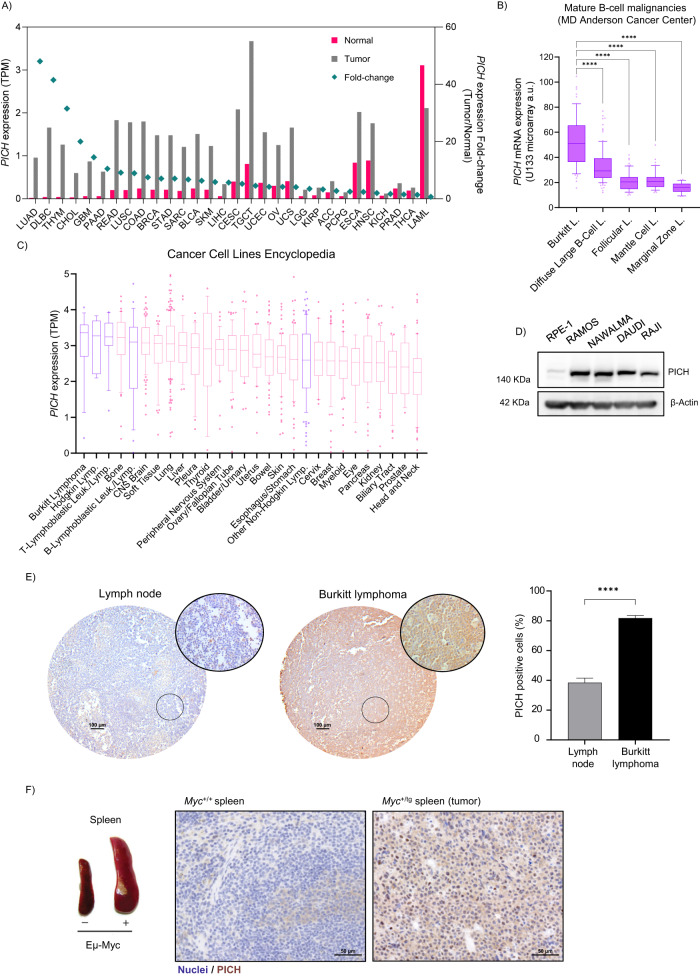
PICH is overexpressed in Burkitt Lymphoma. **A** *PICH* mRNA expression (TPM, Transcripts per million) in the different cancer types from The Cancer Genome Atlas PanCancer Study and paired normal tissue (Adapted from GEPIA). Fold-change in *PICH* expression in tumors compared to normal tissue is shown in the right y-axis. Studies are ranked by fold-change. The study acronyms are LUAD: Lung adenocarcinoma, DLBC Lymphoid Neoplasm Diffuse Large B-cell Lymphoma, THYM Thymoma, CHOL Cholangiocarcinoma, GBM Glioblastoma multiforme, PAAD Pancreatic adenocarcinoma, READ Rectum adenocarcinoma, LUSC Lung squamous cell carcinoma, COAD Colon adenocarcinoma, BRCA Breast invasive carcinoma, STAD Stomach adenocarcinoma, SARC Sarcoma, BLCA Bladder Urothelial Carcinoma, SKCM Skin Cutaneous Melanoma, LIHC Liver hepatocellular carcinoma, CESC Cervical squamous cell carcinoma and endocervical adenocarcinoma, TGCT Testicular Germ Cell Tumors, UCEC Uterine Corpus Endometrial Carcinoma, OV Ovarian serous cystadenocarcinoma, UCS Uterine Carcinosarcoma, LGG Brain Lower Grade Glioma, KIRP Kidney renal papillary cell carcinoma, ACC Adrenocortical carcinoma, PCPG Pheochromocytoma and Paraganglioma, ESCA Esophageal carcinoma, HNSC Head and Neck squamous cell carcinoma, KICH Kidney Chromophobe, PRAD Prostate adenocarcinoma, THCA Thyroid carcinoma, LAML Acute Myeloid Leukemia. **B** Box plot of *PICH* mRNA expression in different lymphoma subtypes in the Mature B-cell malignancies study from MD Anderson Cancer Center. Boxplot shows 10–90 percentile. *n* = 60 (Burkitt Lymphoma), *n* = 95 (Diffuse Large B-cell Lymphoma), *n* = 65 (Follicular Lymphoma), *n* = 43 (Mantle Cell Lymphoma), and *n* = 23 (Marginal Zone Lymphoma). Mean and SEMs are indicated. The levels of PICH expression are significantly higher in BL than in the other four lymphoma subtypes (Diffuse Large B-Cell L., Follicular L., Mantle Cell L., and Marginal Zone L.). Significance was assessed by Tukey’s multiple comparisons test. ****p ≤ 0.0001. The levels are also significantly higher in Diffuse Large B-Cell L. than in Follicular L., Mantle Cell L., and Marginal Zone L. with a *P*-value ****p ≤ 0.0001 with Tukey’s multiple comparisons test. **C** *PICH* expression in human cancer cell lines from the Cancer Cell Line Encyclopedia, adapted from DepMap. Cell lines are grouped by lineage and subtypes are shown for the lymphoid lineage. Boxplot shows 10–90 percentile. **D** Representative immunoblots of PICH protein levels in BL cell lines and the primary non-tumor cell line RPE-1. β-Actin was used as a control for protein load. **E** Representative pictures (left) and quantification (right) of PICH expression in BL and normal lymph node tissue array assessed by IHC staining. Scale bars are indicated, 100 μm. Quantification of the percentage of PICH-positive cells is shown on the right. Data shown correspond to six fields per tissue, counting at least 1000 cells per field. Four normal lymph nodes and three BL tumors were assessed. Means and SEMs are indicated. Significance was assessed by unpaired *t*-test ****p ≤ 0.0001. **F** Representative images of spleens collected from Myc wild-type (*Myc*^+/+^) and Emu-Myc transgenic (*Myc*^+/tg^) mice (left) and immunohistochemical staining against Pich on paraffin sections of spleens from mice with the indicated genotypes (right). Scale bar, 50 μm.

**Fig. 2:**
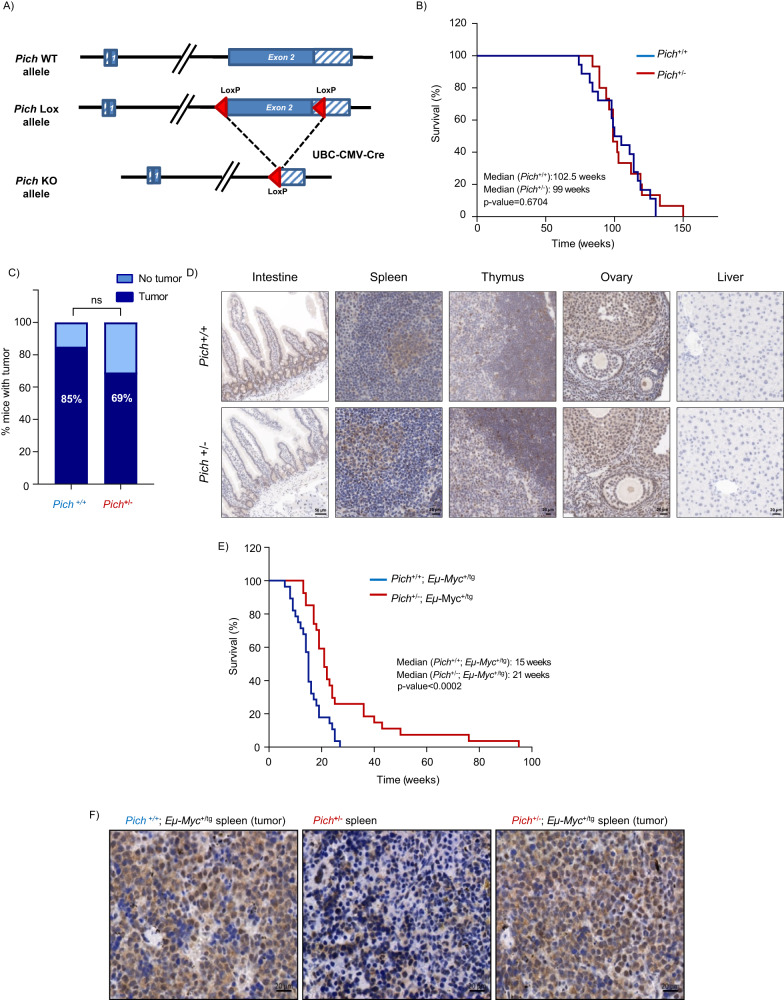
Constitutive PICH deficiency in heterozygosity delays B-cell lymphoma development of *Eµ-Myc* mice. **A** Schematic representation of the alleles of the constitutive *Pich*-deficient mouse model. Two LoxP sites were inserted flanking the endogenous exon 2 of the *Pich* locus to generate *Pich* conditional KO (*Pich*^Lox^) mESC. This *Pich*^Lox^ allele was later combined with the UBC-CMV-Cre allele expressing the Cre recombinase to generate mice harboring the *Pich* KO allele. Coding exons are indicated by filled boxes, and UTR are indicated by hatched boxes. *LoxP* sites are depicted as red triangles. **B** Kaplan–Meier survival curves for *Pich*^+/+^ (*n* = 18; blue; median survival 102.5 weeks) and *Pich*^+/-^ (*n* = 15; red; median survival 99 weeks) mice. *P*-value = 0.6704 using the log-rank (Mantel–Cox) test. **C** Incidence of spontaneous tumors in *Pich*^+/-^ mice compared to *Pich*^+/+^ littermates observed for 150 weeks. The differences observed in tumor incidence were not statistically significant according to the *χ*^2^ test. **D** PICH IHC staining of the indicated tissue sections collected from 50-week-old *Pich*^+/+^ and *Pich*^+/-^ mice. PICH is present in cells of the intestine, spleen, thymus and ovary of both *Pich*^+/+^ and *Pich*^+/-^ mice, but absent in the liver. Scale bar, 50 μm. **E** Kaplan–Meier survival curves for *Pich*^+/+^; *Eµ-Myc*^+/tg^ (*n* = 28; blue; median survival 15 weeks), and *Pich*^+/-^; *Eµ-Myc*^+/tg^ (*n* = 27; red; median survival 21 weeks) mice. *P*-value = 0.0003 using the log-rank (Mantel–Cox) test; ***p ≤ 0.001. **F** Immunohistochemical staining against Pich on spleen or tumoral spleen paraffin sections from *Pich*^+/+^; *Eµ-Myc*^+/tg^, *Pich*^+/+^ and *Pich*^+/-^; *Eµ-Myc*^+/tg^ mice (from left to right). Scale bar, 20 μm.

To investigate the clinical relevance of PICH in human cancer, we evaluated *PICH* mRNA expression in different human cancer tissues and cell lines. We compared *PICH* mRNA expression in the different cancer types from The Cancer Genome Atlas PanCancer Study and their paired normal tissues. *PICH* mRNA expression was elevated in multiple malignancies, including myeloid and lymphoid neoplasms, compared with normal tissues (Fig. 1A). PICH showed significantly higher expression in BL among different lymphoma subtypes (Fig. 1B). Accordingly, when analyzing RNA expression data from the Cancer Cell Line Encyclopedia (CCLE) database which consisted of 1,379 cell lines from a diversity of human tissues, *PICH* mRNA levels were higher in cancer cells from tumors of hematopoietic and lymphoid tissues and, particularly, the highest PICH expression was found in BL (Fig. 1C). BL cell lines also exhibited higher PICH protein expression levels than the non-cancerous and non-transformed human retinal pigment epithelial-1 (RPE-1) cell line (Fig. 1D). Moreover, we performed PICH immunohistochemistry (IHC) analysis of clinical samples from lymphoma and normal lymph nodes, and from mouse spleen and tumors derived from the Eμ-Myc mouse model (Jax strain 2728). Again, human cancer samples showed higher PICH expression compared to normal lymph nodes (Fig. 1E). Mouse spleen tumors derived from the Eμ-Myc mouse model, which develops Myc-induced B-cell lymphoma, also exhibited high PICH expression, in contrast to its lower expression in healthy spleen (Fig. 1F) and in most mouse adult tissues (Fig. 2D). PICH is located in the cytosol of cells in interphase, and its presence on chromatin is restricted to mitosis. As expected, PICH IHC revealed a predominantly cytosolic pattern of PICH localization in interphase cells. Based on these data, we hypothesized that PICH may have a relevant role in BL and that Eμ-Myc mice are a relevant model to investigate this in vivo.

### Pich deficiency in heterozygosity does not induce cancer

To investigate the impact of PICH deficiency in cancer, we used a Pich-deficient mouse model previously generated in our lab [14]. This model consists of a *Pich* conditional KO mouse model with two loxP sites flanking the exon 2 of the murine *Pich* gene. *Pich*^+/Lox^ females were combined with UBC-CMV-Cre mice to generate mice harboring a *Pich* KO allele (Fig. 2A). Whereas complete *Pich* depletion is embryonic lethal by 13.5 days of embryonic development, *Pich* heterozygous mice (*Pich*^+/-^) are viable and do not show any obvious abnormalities [14]. Given that *Pich* is located in the X chromosome, only heterozygous females could be investigated, thus, only female mice were included in the cohorts. To test the long-term effects of Pich deficiency, we evaluated the tumor-free survival and spontaneous tumor formation of *Pich*^+/+^ and *Pich*^+/-^ mice for up to 150 weeks. Mice were euthanized when they had noticeable tumors or were visibly ill, as observed by rapid weight loss, hunched posture, rough hair coat, labored breathing, lethargy, impaired mobility or abdominal swelling. Females from both cohorts survived at least 74 weeks and did not show significant differences in their median tumor-free survival rates (Fig. 2B). All *Pich*^+/-^ mice tested remained healthy and did not show weight loss or behavioral changes during this time. In addition, the percentage of mice that developed spontaneous tumors at the end of the experiment was similar in both *Pich*^+/+^ and *Pich*^+/-^ mice (Fig. 2C). We did not find differences in the number of spontaneous tumors per mouse nor in the tumor types between both cohorts (Supplementary Fig. 1). Histological examination of tissues collected from 50-week-old *Pich*^+/-^ mice (not included in the survival curve) did not reveal structural abnormalities (Fig. 2D). Analysis of adult tissues from *Pich*^+/+^ mice confirmed high PICH expression in tissues with an elevated percentage of proliferating cells, including those of the intestines, spleen, thymus, and ovaries in contrast to the liver that do not express PICH at detectable levels. Interestingly, *Pich*^+/-^ mice exhibit similar PICH expression patterns to *Pich*^+/+^ females during adulthood (Fig. 2D). Overall, these results indicate that *Pich* heterozygosity does not promote tumorigenesis in vivo nor have negative effects on the physiology of healthy mice.

### Pich deficiency in heterozygosity delays the onset of tumors in Eμ-Myc mice

To investigate the effect of Pich depletion in *Myc*-induced lymphoma development in vivo, *Pich* heterozygous females were crossed with the transgenic Eμ-Myc cancer mouse model. In this tumor model, *c-Myc* gene expression is driven by the IgH enhancer and is expressed in B-lymphoid cells, which mimics the translocated *Myc* genes in B-cell lymphoma. These mice spontaneously develop B-cell lymphoma with 100% penetrance and a median latency of 16–20 weeks [33]. We evaluated the survival of *Pich*^+/+^ and *Pich*^+/-^ females in the Eµ-myc transgenic background. Pich deficiency, in heterozygosity, significantly delayed the onset of *Myc*-induced lymphoma (Fig. 2E). While the control cohort of *Pich*^+/+^; *Eµ-Myc*^+/tg^ mice had a median survival rate of 15 weeks, *Pich*^+/-^; *Eµ-Myc*^+/tg^ mice had a median survival rate of 21 weeks. The median survival was increased by 50%, with some *Pich*^+/-^ mice surviving up to 90 weeks of age (Fig. 2E).

PICH expression was analyzed by IHC in tumors derived from *Pich*^+/-^; *Eµ-Myc*^+/tg^ mice. Remarkably, we observed a homogeneous PICH expression in contrast to the patchy pattern present in tissues from *Pich*^+/-^ mice and similar to the one found in *Pich*^+/+^; *Eµ-Myc*^+/tg^ BL tumors (Fig. 2F). Note that *Pich* is located on the X chromosome and, therefore, heterozygous females are expected to exhibit mosaic PICH expression. Based on these results, X-chromosome inactivation (XCI) seems to be biased towards the allele containing the *Pich* KO allele in *Pich*^+/-^; *Eµ-Myc*^+/tg^ cancer cells, suggesting that PICH is necessary for the development of these tumors.

### *Pich* deletion limits *Myc*-induced lymphoma progression

**Fig. 3:**
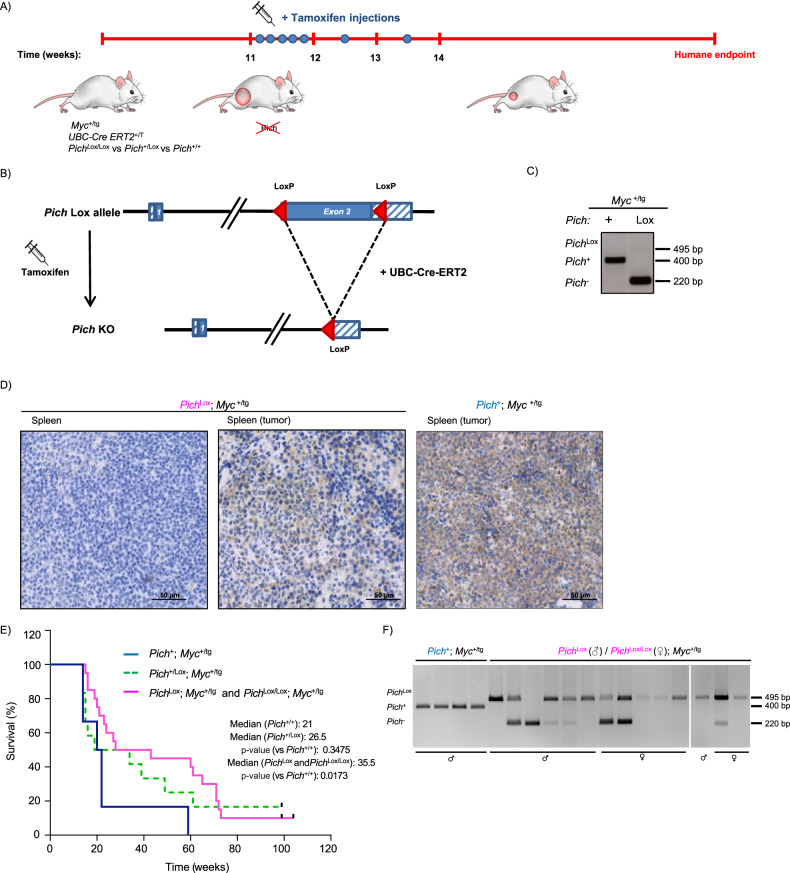
PICH depletion limits the progression of MYC-induced B-cell lymphoma. **A** Experimental overview to test the effect of deleting *Pich* in lymphoma progression. *Myc*^+/tg^; *Pich*^+^; *UBC-Cre-ERT2*^+/T^, *Myc*^+/tg^; *Pich*^+/Lox^; *UBC-Cre-ERT2*^+/T^, and *Myc*^+/tg^; *Pich*^Lox^; *UBC-Cre-ERT2*^+/T^ or *Myc*^+/tg^; *Pich*^Lox/Lox^; *UBC-Cre-ERT2*^+/T^ mice were generated. Eleven-week-old mice were injected with tamoxifen daily for a total of 5 consecutive days, plus 2 repeated injections for the next 2 weeks to induce *Pich* deletion and monitored for survival. **B** Representation of the deletion of the *Pich* allele in the *Pich* conditional KO mouse model. Recombination of the floxed conditional allele of *Pich* after UBC-Cre-ERT2 activation upon tamoxifen treatment. Coding exons are indicated by filled boxes, and UTR regions are indicated by hatched boxes. *LoxP* sites are depicted as red triangles. **C** Genomic PCR analysis of *Pich*^Lox^ allele recombination after treatment with tamoxifen. Genotyping PCR analysis from genomic DNA isolated from clip tails of tamoxifen-treated *Myc*^+/tg^; *Pich*^+^; *UBC-Cre-ERT2*^+/T^, and *Myc*^+/tg^; *Pich*^Lox^; *UBC-Cre-ERT2*^+/T^ mice collected at humane endpoint. Migration of the expected unrecombined *Pich*^Lox^ allele (495 bp), the wild-type *Pich*^*+*^ allele (440 bp) and the ablated *Pich*^*-*^ allele (220 bp) are indicated. **D** PICH IHC staining of tissue sections collected from the indicated tamoxifen-treated mice. Left panel: non-tumoral spleen from healthy *Myc*^+/tg^; *Pich*^Lox^; *UBC-Cre-ERT2*^+/T^ mice 20 weeks after tamoxifen treatment. Central panel: tumoral spleen collected from tamoxifen-treated *Myc*^+/tg^; *Pich*^Lox^; *UBC-Cre-ERT2*^+/T^ mice at humane endpoint. Right panel: tumoral spleen collected from tamoxifen-treated *Myc*^+/tg^; *Pich*^+^; *UBC-Cre-ERT2*^+/T^ mice at humane endpoint. **E** Kaplan–Meier survival curves for *Myc*^+/tg^; *Pich*^+^; *UBC-Cre-ERT2*^+/T^ (*n* = 6; blue; median survival 21 weeks), *Myc*^+/tg^; *Pich*^+/Lox^; *UBC-Cre-ERT2*^+/T^ (*n* = 13; hatched green; median survival 26.5 weeks) and *Myc*^+/tg^; *Pich*^Lox^; *UBC-Cre-ERT2*^+/T^ or *Myc*^+/tg^; *Pich*^Lox/Lox^; *UBC-Cre-ERT2*^+/T^ (*n* = 20; pink; median survival 35.5 weeks) mice treated with tamoxifen at 11 weeks of age. Black dots indicate mice alive at the end of the experiment. The log-rank (Mantel–Cox) test was performed to assess differences in survival of *Pich*^Lox^ or *Pich*^Lox/Lox^ (*p* = 0.0173) and *Pich*^+/Lox^ (*p* = 0.3475) mice compared to *Pich*^+/+^ mice, respectively. **F** *Pich* genotyping PCR analysis from genomic DNA isolated from individual tumors of *Myc* ^+/tg^; *Pich*^+^; *UBC-Cre-ERT2*^+/T^, *Myc*^+/tg^; *Pich*^Lox^; *UBC-Cre-ERT2*^+/T^ and *Myc*^+/tg^; *Pich*^Lox/Lox^; *UBC-Cre-ERT2*^+/T^ mice at the humane endpoint. Each lane corresponds to a tumor from an individual mouse. The gender of each mouse is indicated in the figure. Migration of the expected unrecombined *Pich*^Lox^ allele (495 bp), the wild-type *Pich*^*+*^ allele (440 bp) and the ablated *Pich*^*-*^ allele (220 bp) are indicated.

We next investigated the effect of PICH depletion in *Myc*-induced B-cell lymphoma progression. To this end, we generated *Myc*^+/tg^; *Pich*^Lox/Lox^; *UBC-Cre-ERT2*^+/T^ mice. *UBC-Cre-ERT2* mice (Jax strain 007001) ubiquitously express an inducible Cre-ERT2 recombinase, selectively activated only in the presence of 4-hydroxytamoxifen, a metabolite of the prodrug tamoxifen [34]. In combination with the Lox-conditional allele of *Pich*, the UBC-Cre-ERT2 system allows efficient *Pich* deletion in all tissues of adult mice upon tamoxifen treatment. *Pich* deletion was induced at 11 weeks of age, when formation of tumors is expected to be underway. Both females and males with *Pich*^Lox/Lox^ and *Pich*^Lox^ genotypes, respectively, were included in the cohorts. Eleven-week-old *Myc*^+/tg^; *Pich*^Lox^; *UBC-Cre-ERT2*^+/T^ and *Myc*^+/tg^; *Pich*^Lox/Lox^; *UBC-Cre-ERT2*^+/T^ mice, along with *Myc*^+/tg^; *Pich*^+/Lox^; *UBC-Cre-ERT2*^+/T^ and control animals (*Myc*^+/tg^; *Pich*^+^; *UBC-Cre-ERT2*^+/T^), were injected with tamoxifen into the peritoneum once every 24 h for a total of 5 consecutive days, plus 2 repeated injections the next 2 weeks (Fig. 3A). Activation of the UBC-Cre-ERT2 by tamoxifen treatment led to efficient recombination of the *Pich* locus and reduced PICH protein expression in non-tumoral tissues from *Myc*^+/tg^; *Pich*^Lox^; *UBC-Cre-ERT2*^+/T^ and *Myc*^+/tg^; *Pich*^Lox/Lox^; *UBC-Cre-ERT2*^+/T^ (Fig. 3B–D (left panel)). Survival of tamoxifen-treated *Myc*^+/tg^; *Pich*^Lox^; *UBC-Cre-ERT2*^+/T^ and *Myc*^+/tg^; *Pich*^Lox/Lox^; *UBC-Cre-ERT2*^+/T^ mice was increased compared to control mice (median survival from 21 to 35.5 weeks) (Fig. 3E). In addition, *Myc*^+/tg^; *Pich*^+/Lox^; *UBC-Cre-ERT2*^+/T^ mice treated with tamoxifen also displayed intermediate increased survival. Thus, PICH seems to be required to sustain *Myc*-induced lymphoma, and the proliferation capacity of these tumors is diminished upon *Pich* deletion, which impacts the survival of the mice.

We also analyzed PICH expression in tumors derived from the mice sacrificed at the designated humane endpoint (determined by rapid weight loss, hunched posture, rough hair coat, labored breathing, lethargy, impaired mobility or abdominal swelling) and compared it to non-tumoral spleens. Most of the Pich expression was lost in non-tumoral spleens expressing the *Eµ-Myc* allele that presented a normal histology upon the induction of *Pich* deletion with tamoxifen (Fig. 3D, left panel). In contrast, *Myc*^+/tg^; *Pich*^Lox^; *UBC-Cre-ERT2*^+/T^ and *Myc*^+/tg^; *Pich*^Lox/Lox^; *UBC-Cre-ERT2*^+/T^ tumors exhibited a high percentage of cells with positive Pich staining (Fig. 3D, central panel). Moreover, analysis of genomic DNA isolated from these tumors revealed the presence of unrecombined *Pich*^Lox^ alleles (Fig. 3F). Thus, Pich-positive tumor cells detected in the tumorigenic spleen by Pich staining result from the partial recombination of the *Pich*^Lox^ allele, indicating that tumors have a preference for PICH expression to develop and hence there is a tumoral selective pressure for retaining PICH expression. Nevertheless, the recombination of the *Pich*^Lox^ allele achieved in the mice of our study was sufficient to extend the survival of the mice. These observations reinforce the hypothesis that Pich is important for B-cell lymphoma progression. Interestingly, we identified one lymphoma with an efficient recombination of the *Pich* locus, suggesting that PICH is not completely essential and other factors may compensate for its absence in some cases.

**Fig. 4:**
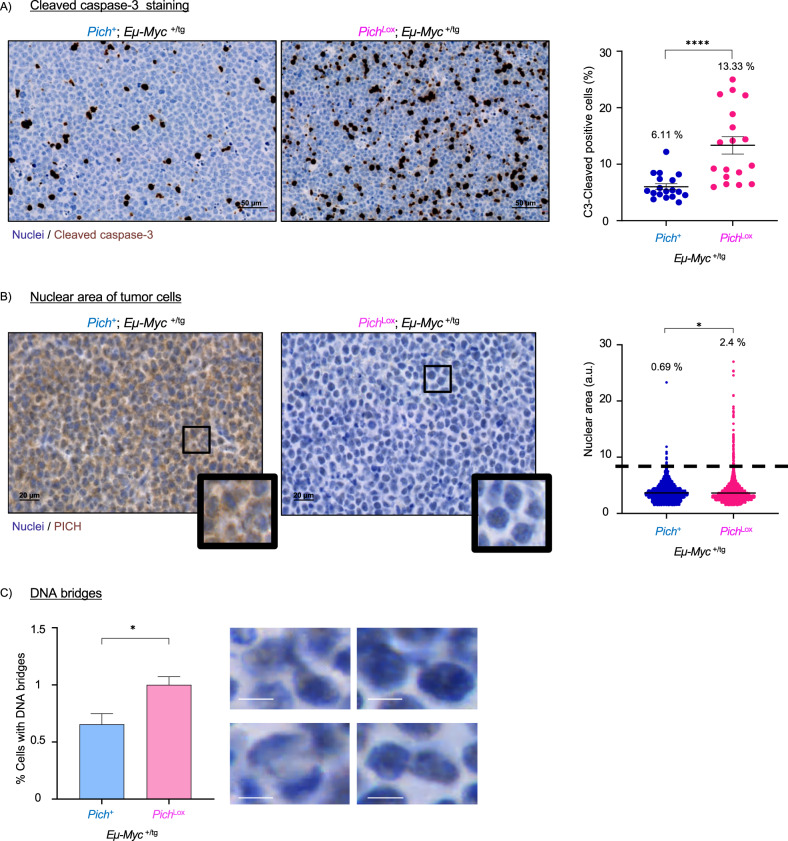
B-cell lymphoma tumors from tamoxifen-treated MYC-induced mice exhibit increased apoptosis and nuclear volume. **A** Representative immunohistochemical staining using anti-cleaved Caspase-3 antibody of paraffin-fixed tumor sections obtained at humane end point of *Myc* ^+/tg^; *Pich*^+^; *UBC-Cre-ERT2*^+/T^ (left) and *Myc* ^+/tg^; *Pich*^Lox^; *UBC-Cre-ERT2*^+/T^ (right) mice treated with tamoxifen. Scale bar, 50 μm. Quantification of the percentage of cleaved caspase-3 positive cells is shown on the right. Points represent the percentage of cleaved caspase-3 positive cells on each field. Data shown correspond to four to six fields per tumor, counting at least 800 cells per field. Tumors from four *Pich* wild-type and three *Pich* KO mice were assessed. Numbers indicate the mean percentage of cleaved caspase-3 positive cells. Means and SEMs are indicated. Significance was assessed by unpaired *t*-test ****p ≤ 0.0001. **B** Representative immunohistochemical staining using anti-PICH antibody of paraffin-fixed tumor sections obtained at humane endpoint of *Pich*^+^; *Eµ-Myc*^+/tg^; *UBC-Cre-ERT2*^+/T^ (left) and *Pich*^Lox^; *Eµ-Myc*^+/tg^; *UBC-Cre-ERT2*^+/T^ (right) treated with tamoxifen. Scale bar, 20 μm. Quantification of the nuclear area is shown on the right. Points represent individual nuclei. Data shown correspond to four to six fields per tumor, counting at least 400 cells per field. Tumors from three *Pich* wild-type and three *Pich* KO mice were assessed. Numbers indicate the percentage of cells with a nuclear area larger than 8 arbitrary units (a.u.). Cells with a nuclear area at least double that of the mean of cells from *Myc* ^+/tg^; *Pich*^+^; *UBC-Cre-ERT2*^+/T^ animals (3.7). Means and SEMs are indicated. Significance was assessed by unpaired *t*-test. *p ≤ 0.05. **C** B-cell lymphoma tumors from Pich-deficient tamoxifen-treated *Myc*-induced mice exhibit increased percentage of cells with DNA bridges. Quantification of the percentage of cells with DNA bridges is shown on the left. Data shown correspond to four to six fields per tumor, counting at least 400 cells per field. Tumors from three *Myc* ^+/tg^; *Pich*^+^; *UBC-Cre-ERT2*^+/T^ and three *Myc* ^+/tg^; *Pich*^Lox^; *UBC-Cre-ERT2*^+/T^ tamoxifen-treated mice obtained at humane endpoint were assessed. Means and SEMs are indicated. Significance was assessed by unpaired *t*-test. *p ≤ 0.05. Representative images of DNA bridges from Pich-deficient tumors are shown on the right. Scale bar, 5 μm.

Next, we investigated the mechanisms that contributed to the reduced *Myc*-induced tumor progression observed in Pich-depleted mice. We first evaluated DNA damage and apoptosis in tissues from tamoxifen-treated tumors at the designated humane endpoint. Of note, tumors with total or partial recombination of the *Pich* Lox allele were used for these analyses. A high number of apoptotic cells in tumors from *Pich*-depleted mice compared to tumors for mice with normal Pich expression was observed (Fig. 4A), as determined by IHC staining for cleaved caspase-3. However, the number of tumor cells positive for ϒ-H2AX, a marker of DNA damage was similar in both mice (data not shown). It has been shown that PICH deficiency results in abnormal chromosome segregation and cytokinesis, generating polyploid and binucleated cells [12]. This is supported by the observation that these cells exhibit a larger nuclear area compared to euploid cells, which has been shown to correlate with increased ploidy [35, 36]. Similarly, Pich depletion increased the percentage of tumoral cells with higher nuclear area in our mouse model, suggesting an increase of polyploid cells (Fig. 4B). Additionally, we examined the presence of DNA bridges in Pich-deficient tumors in the hematoxylin/eosin stainings. A higher percentage of cells with apparent DNA bridges was observed in tumors from tamoxifen-treated *Myc*^+/tg^; *Pich*^Lox^; *UBC-Cre-ERT2*^+/T^ and *Myc*^+/tg^; *Pich*^Lox/Lox^; *UBC-Cre-ERT2*^+/-^ mice compared to tumors from *Pich* wild-type counterparts (Fig. 4C).

Taken together, these results demonstrate that *Pich* ablation in *Myc*-induced mice gives rise to defects in chromosomal segregation and chromosomal instability that result in increased apoptosis and a reduction in proliferative capacity, which ultimately limits tumor growth.

### Systemic depletion of PICH in adult mice is well tolerated

**Fig. 5:**
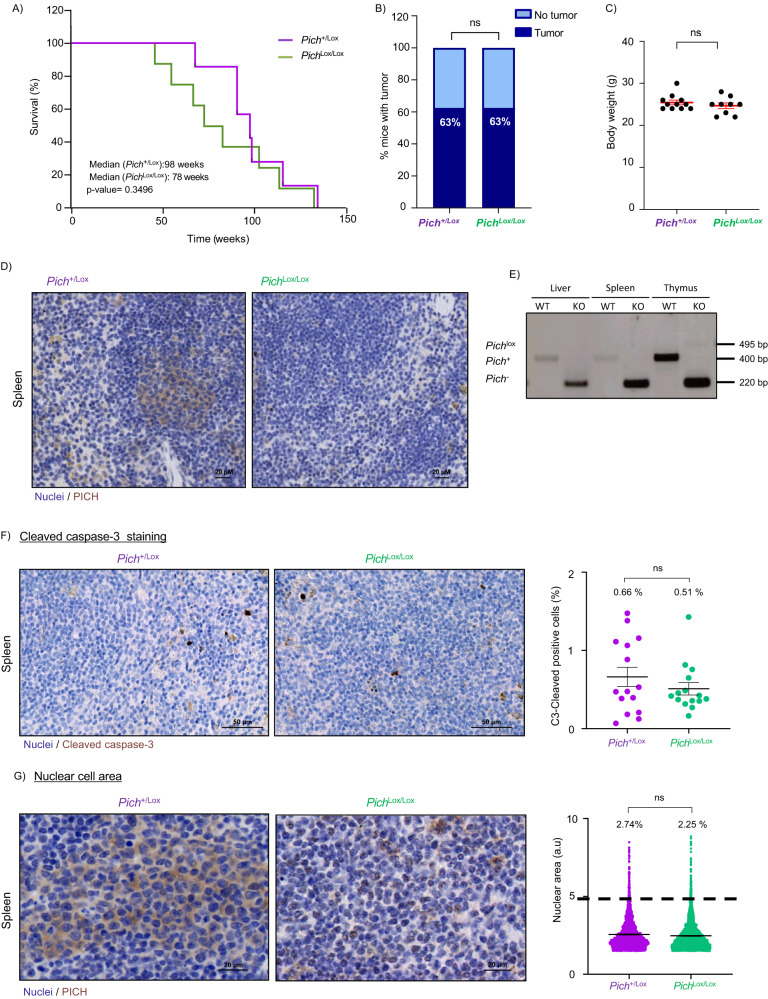
Systemic PICH depletion is compatible with normal homeostasis in adult mice. **A** Kaplan–Meier survival curves for tamoxifen-treated *Pich*^+/Lox^; *UBC-Cre-ERT2*^+/T^ (*n* = 8; green; median survival 98 weeks) and *Pich*^Lox/Lox^; *UBC-Cre-ERT2*^+/T^ (*n* = 8; purple; median survival 78 weeks) mice. *P*-value = 0.3496 using the log-rank (Mantel–Cox) test. **B** Incidence of spontaneous tumors in *Pich*^+/Lox^; *UBC-Cre-ERT2*^+/T^ mice compared to *Pich*^Lox/Lox^; *UBC-Cre-ERT2*^+/T^ littermates treated with tamoxifen at eleven weeks of age. The differences observed in tumor incidence were not statistically significant according to the log-rank (Mantel–Cox) test. **C** Total body weights of adult mice at one year of age. Means and SEMs are indicated. Significance was assessed by unpaired *t*-test. *P*-value = 0.3746. **D** Analysis of *Pich*^Lox^ allele recombination in tamoxifen-treated *Pich* conditional KO mice. PICH IHC staining of spleen tissue sections collected from 1-year-old *Pich*^+/Lox^; *UBC-Cre-ERT2*^+/T^ and *Pich*^Lox/Lox^; *UBC-Cre-ERT2*^+/T^ mice. Scale bar, 20 μm. Tissues from six mice were extracted for anatomical examination. **E** *Pich* genotyping by PCR of genomic DNA isolated from individual tissues of *Pich*^+/Lox^; *UBC-Cre-ERT2*^+/T^ and *Pich*^Lox/Lox^; *UBC-Cre-ERT2*^+/T^ mice at humane endpoint. Migration of the expected unrecombined *Pich*^Lox^ allele (495 bp), the wild-type *Pich*^+^ allele (440 bp) and the ablated *Pich*^-^ allele (220 bp) is indicated. **F** Representative immunohistochemical staining using anti-cleaved Caspase-3 antibody of fixed spleen sections obtained from *Pich*^+/Lox^; *UBC-Cre-ERT2*^+/T^ (left) and *Pich*^Lox/Lox^; *UBC-Cre-ERT2*^+/T^ littermates 33 weeks after tamoxifen treatment. Scale bar, 50 μm. Quantification of the percentage of cleaved caspase-3 positive cells is shown on the right. Points represent the percentage of cleaved caspase-3 positive cells on each field. Data shown correspond to four to six fields per spleen, counting at least 800 cells per field. Normal proliferative spleen for four *Pich*^+/Lox^ and four *Pich*^Lox/Lox^ healthy mice were assessed. Numbers indicate the mean percentage of cleaved caspase-3 positive cells. Means and SEMs are indicated. The differences observed were not statistically significant according to the unpaired *t*-test (*p* ≤ 0.05). **G** Representative immunohistochemical staining using anti-PICH antibody of fixed spleen sections obtained from *Pich*^+/Lox^; *UBC-Cre-ERT2*^+/T^ and *Pich*^Lox/Lox^; *UBC-Cre-ERT2*^+/T^ littermates 33 weeks after tamoxifen treatment. Scale bar, 20 μm. Quantification of the nuclear area is shown on the right. Points represent individual nuclei. Data shown correspond to four to six fields per spleen, counting at least 500 cells per field. Normal proliferative spleen from four *Pich*^+/Lox^ and four *Pich*^Lox/Lox^ healthy mice were assessed. Numbers indicate the percentage of cells with a nuclear area larger than 5 arbitrary units (a.u.). Cells with a nuclear area at least double that of the mean of cells from *Pich*^+/Lox^; *UBC-Cre-ERT2*^+/T^ animals (2.5). Means and SEMs are indicated. The differences observed were not statistically significant according to the unpaired *t*-test (*p* ≤ 0.05).

Tumor-selective cancer treatments are urgently needed to avoid the deleterious consequences on healthy tissues, which cause toxic side effects in patients. One of the strategies to achieve tumor selectivity is the design of drugs that target pathways essential for the survival of cancer cells, but not normal cells. In this line, we examined whether Pich was required for normal homeostasis in adult mice. In previous experiments, *Pich* deletion in *Myc*^+/tg^; *Pich*^Lox/Lox^; *UBC-Cre-ERT2*^+/-^ mice was induced in all tissues and did not cause apparent systemic alterations. However, since these mice develop BL and die at a young age (median 35.5 weeks, Fig. 3E), we wanted to investigate the potential longer-term consequences of PICH deletion in adult tissues. At the age of 11 weeks*, Pich*^+/Lox^; *UBC-Cre-ERT2*^+/-^ or *Pich*^Lox/Lox^; *UBC-Cre-ERT2*^+/-^ mice were treated with tamoxifen as described above and monitored until they had noticeable tumors, reached the designated humane endpoint or up to 135 weeks. Survival of *Pich*^Lox/Lox^; *UBC-Cre-ERT2*^+/-^ and control mice was similar (Fig. 5A). With regard to spontaneous tumor formation, none of the tamoxifen-treated mice developed spontaneous tumors before 47 weeks of age (Fig. 5B), and the percentage of mice that developed tumors was similar in both cohorts (Fig. 5B). In addition, we observed no noticeable differences in appearance among the mice with different genotypes, as reflected by their weight at 1 year of age (Fig. 5C).

Tissues from tamoxifen-treated mice were extracted for histological examination and to ascertain recombination efficiency of the *Pich*^Lox^ allele, at 1, 6, and 52 weeks after tamoxifen treatment. *Pich*^Lox/Lox^; *UBC-Cre-ERT2*^+/-^ tissues displayed efficient recombination of the *Pich*^Lox^ allele, as assessed by IHC (Fig. 5D and Supplementary Fig. 2A). The presence of the recombined *Pich* KO allele in the genomic DNA was also confirmed by PCR in all tissues tested (Fig. 5E and Supplementary Fig. 2B). The histological analysis did not reveal histological differences between the organs of one-year-old tamoxifen-treated *Pich*^Lox/Lox^; *UBC-Cre-ERT2*^+/-^ mice and those of control animals, with all exhibiting normal architecture (Fig. 5D and Supplementary Fig. 2A). In addition, *Pich* deletion in spleen and intestines did not lead to apoptosis, determined by IHC staining for cleaved caspase-3 (Fig. 5F and Supplementary Fig. 2C), nor changes in the number of polyploid cells (Fig. 5G and Supplementary Fig. 2D).

Overall, these findings demonstrate that systemic PICH deletion in adult mice is well tolerated and does not cause major toxic effects. Hence, PICH seems to be essential for tumor progression but not for cells of healthy adult organs, which places PICH as a promising therapeutic target.

### PICH knockdown inhibits proliferation and induces cell death in human BL cells

**Fig. 6:**
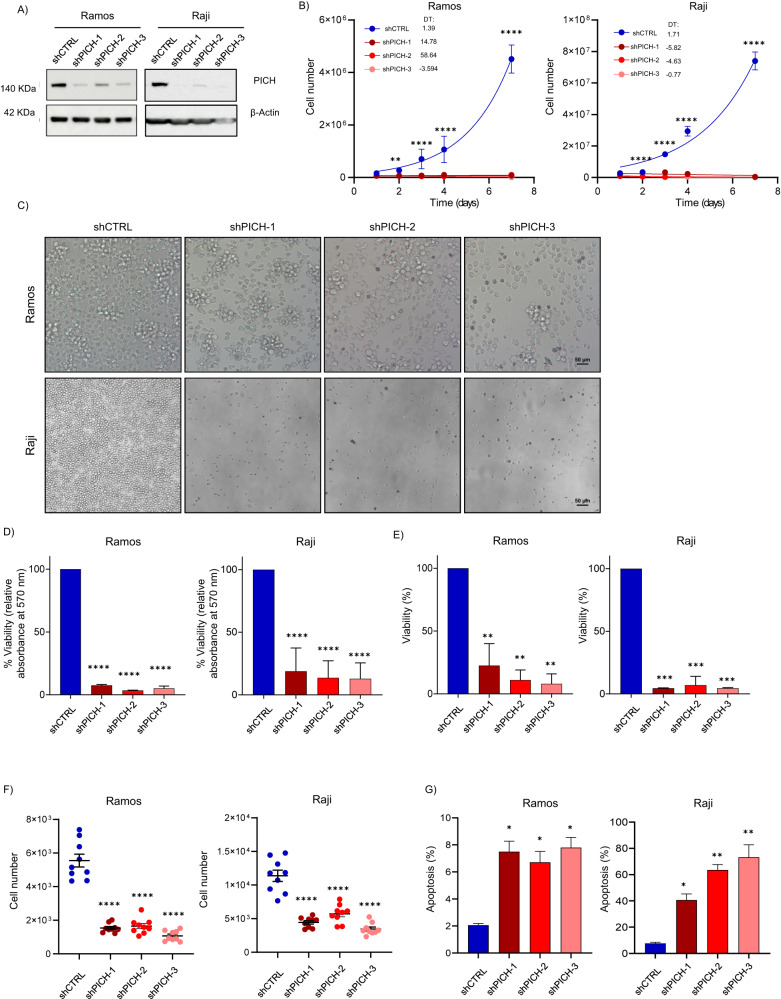
PICH knockdown decreases proliferation and induces cell death in Ramos and Raji human BL cell lines. **A** Representative immunoblots of PICH protein levels in Ramos (left) and Raji (right) BL cells seven days after transduction with lentiviruses expressing the indicated shPICH or shControl (shCTRL). β-Actin was used as loading control. **B** Cell proliferation curve of Ramos (left) and Raji BL cells (right) infected with shCTRL or the indicated shPICH. PICH KD cells proliferated at a lower rate than shCTRL cells and stopped proliferating after four passages. Data shown correspond to a representative experiment (with technical triplicates) out of three biological replicates. The corresponding doubling times (DT) were calculated by non-linear regression and are shown in each graph. SEMs from each data point are indicated and the *p*-value for each dataset is shown. **p < 0.01; ****p < 0.0001. **C** Representative images of Ramos (top) and Raji (bottom) BL cells 10 days after infection with either shCTRL or shPICH. The images were captured at 4x magnification with a light optical microscope. Scale bar, 50 μm. Images were acquired immediately prior to the addition of MTT into the wells. **D** Relative cell viability of Ramos (left) and Raji (right) BL cells upon depletion of PICH compared to transfection with control shRNA (shCTRL) assessed by MTT assay 10 days after infection. Data are represented as relative absorbance at 570 nm and correspond to four independent experiments with six technical replicates for each experiment. Mean and SEMs are indicated. Significance was assessed by unpaired *t*-test. ****p ≤ 0.0001. **E** Relative cell viability of Ramos (left) and Raji (right) BL cells upon depletion of PICH compared to transfection with control shRNA (shCTRL) determined by cell counting after labeling cells with trypan blue 10 days after infection. Histograms show the mean of the number of non-trypan blue-stained live cells of PICH depleted cells relative to shCTRL cells and correspond to three independent experiments with three technical replicates for each experiment. Mean and SEMs are indicated. Significance was assessed by unpaired *t*-test. ***p ≤ 0.001; ****p ≤ 0.0001. **F** Cell viability of Ramos (left) and Raji (right) BL cells 10 days after transduction with lentiviruses expressing the indicated shPICH or shCTRL. Growing cells were incubated with Hoechst 33342 and To-Pro-3 dyes for 30 min. After labeling, images were acquired with a High-Content Screening System (MetaXpress) at 10× magnification. Quantification of Hoechst 33342-positive and To-Pro-3 positive cells were performed with the MetaXpress High-Content Image Analysis Software. Live cells were calculated by subtracting the number of To-Pro-3 positive cells from the Hoechst 33342-positive total cell number. Data correspond to three independent experiments with three technical replicates for each experiment. Mean and SEMs are indicated. Significance was assessed by unpaired *t*-test. ****p ≤ 0.0001. **G** Effect of PICH KD on the apoptosis of Ramos and Raji BL cancer cells measured by Annexin V-FITC/Propidium iodide and flow cytometry 7 days after infection with either shCTRL or shPICH. The percentage of apoptotic (Annexin V-positive) cells is indicated. Mean and SEMs are indicated. Significance was assessed by unpaired *t*-test. *p ≤ 0.05; **p ≤ 0.01.

To determine the relevance of PICH in human BL, we analyzed the consequences of losing PICH in human BL tumoral cells. Three shRNAs were transduced into Ramos and Raji BL cells to deplete PICH expression and the knockdown efficiency was assessed by Western blotting (Fig. 6A and Supplementary Fig. 3A). The effect of PICH knockdown on cell proliferation was measured over 14 days after lentiviral infection. PICH silencing strongly decreased the proliferative capacity of human BL cells (Fig. 6B). This reduction in the proliferation rates was not accompanied by major defects in DNA replication, as assessed by EdU incorporation assay (Supplementary Fig. 3B). Next, we evaluated the impact of PICH deficiency on cell viability by three different techniques, namely the MTT assay (Fig. 6C, D), trypan blue staining (Fig. 6E), and by Hoechst and To-Pro DNA fluorescence staining (Fig. 6F). Targeting *PICH* also led to a strong depletion of BL cell viability. Cell viability was notably reduced in the shPICH cells compared to the control cells (Fig. 6C-F). Next, apoptosis was determined using Annexin V/PI staining eight days after lentivirus infection and an increased number of apoptotic PICH-KD cells compared to cells expressing normal PICH levels were found (Fig. 6G). Thus, the effect of PICH depletion inhibiting BL cell viability was, at least partially, accounted for by the induction of apoptosis.

### PICH depletion causes chromosomal instability in human BL cells

**Fig. 7:**
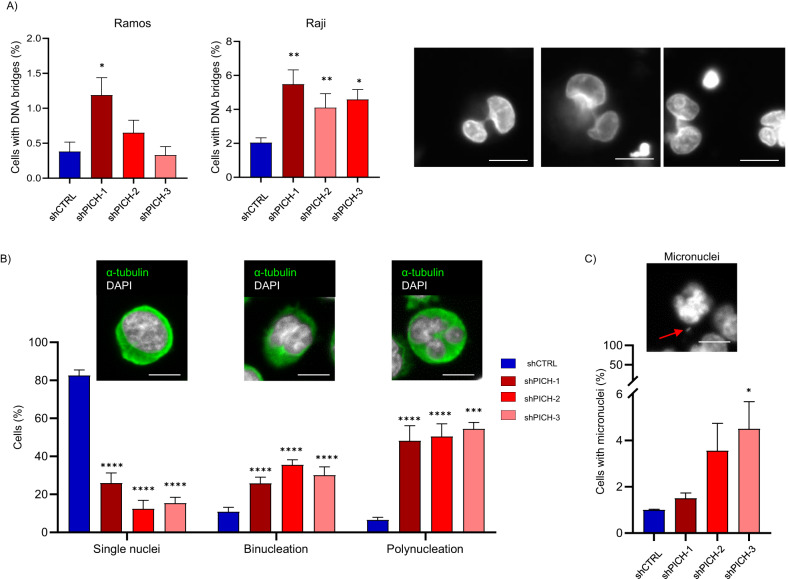
Increased chromosomal instability of PICH-deficient Ramos and Raji human BL cells. **A** Frequency of DNA bridges in asynchronously Ramos and Raji BL growing cells was determined by Hoechst 33342 nuclear staining (white) 7 days after PICH depletion with the indicated shRNAs. After staining, samples were examined with High-Content Screening System MetaXpress at ×40 magnification. Data shown correspond to biological triplicates of at least 300 cells per condition. Mean and SEMs are indicated. Significance was assessed by unpaired *t*-test. *p ≤ 0.05; **p ≤ 0.01. Representative images are shown. Scale bar, 5 μm. **B** Frequency of bi- and multi-nucleation in asynchronously growing Ramos BL cells was determined by immunofluorescence staining of α-tubulin (green) combined with DAPI nuclear staining (white) 7 days upon PICH depletion with shRNAs. After immunostaining, samples were examined with High-Content Screening System MetaXpress at 40× magnification. Data shown correspond to biological triplicates of at least 500 cells per condition. Mean and SEMs are indicated. Significance was assessed by unpaired *t*-test. ***p ≤ 0.001 ****p ≤ 0.0001. Representative images are shown. Scale bar, 5 μm. **C** Frequency of micronucleus formation in cells stained with DAPI and obtained as described in Fig. 7B. Mean and SEMs are indicated. Significance was assessed by unpaired *t*-test. *p ≤ 0.05. An example of a scored micronucleus is shown. Red arrow indicates a micronucleus in the representative image. Scale bar, 5 μm.

To characterize the cause of cell lethality in PICH-depleted BL cells, we analyzed defects in cell division. Abnormal chromosome segregation usually leads to cytokinesis problems and abnormal cell division that is manifested as G1-associated aberrations, such us micronuclei and bi- or poly-nucleation. PICH knockdown in human cells has been shown to increase the occurrence of chromosomal abnormalities [12]. We examined whether silencing *PICH* in BL cells showed an alteration in the frequency of chromosome abnormalities. Of note, proliferative BL Ramos and Raji cell lines PICH positive persistent bridges (Supplementary Fig. 3C). In agreement with the results observed in PICH-deficient Eµ-Myc tumors, PICH-depleted human BL cells exhibited an increased frequency of persistent DNA bridges revealed by Hoechst 33342 staining of nuclei (Fig. 7A). PICH-deficient BL cells also had a significantly elevated frequency of binucleation (cells with two decondensed daughter nuclei in the same plasma membrane), polynucleation (Fig. 7B), and micronucleus formation, revealed by DAPI staining of nuclei and immunofluorescence staining for α-tubulin (Fig. 7C). Altogether, these data suggest that PICH deficiency results in defective cell division in BL cells, thus suppressing cell proliferation and inducing cell death.

## Discussion

PICH is a DNA translocase that plays a role in the resolution of UFBs and is necessary for the maintenance of chromosomal stability in proliferating cells. Previously, we found that PICH is essential for embryonic development and several groups showed that PICH is highly expressed in several cancerous cells and is relevant for their proliferation in vitro [18–25]. Moreover, a recent study has shown that PICH deficiency limits the development of mammary tumors in mice [37]. Given that PICH expression is highest in BL among all cancer types, we have used a mouse model of *Myc*-induced B-cell lymphoma (*Eμ-Myc*), in combination with the *Pich* conditional KO model. We have found that PICH depletion limits the progression of MYC-induced lymphomas and, critically, does not have major toxic effects in non-tumoral tissues.

It is important to stress the contrast between the consequences of PICH depletion during embryonic development, which is incompatible with life, and PICH depletion in adult mice, which is very well tolerated. This is particularly relevant when considering PICH as a potential therapeutic target. Many proteins are required for the proliferation of cancerous cells, but few of them are highly specific for cancerous compared to normal tissues. Thus, we believe that PICH inhibitors may be useful for cancer therapy, and we are currently working on the identification and development of small molecules with PICH inhibitory capacity.

We have also investigated the long-term effect of PICH depletion in adult mice without additional alterations because PICH depletion could potentially lead to chromosomal instability (CIN) and increased cancer risk. However, we did not observe an increased incidence of any cancer type. Indeed, whether CIN itself may lead to cancer or not is still a matter of debate, and may vary between different cellular and genetic contexts [38].

Previous molecular analyses performed by us and other groups [7, 11, 14], and our observations of polyploid cells and chromosomal abnormalities in PICH-deficient cancer cells, suggest that the molecular mechanism by which PICH favors the proliferation of cancer cells is the resolution of UFBs and maintenance of chromosomal stability. However, other studies have reported that PICH also plays an additional role stabilizing the replication fork [39] and regulates the PI3K/AKT and NK- κB pathways [22] that may also contribute to cancer progression. Of note, increased levels of replication stress, such as those caused by MYC [40] overexpression, could lead to the formation of UFBs. It is well characterized that PICH binds to UFBs, where it recruits and modulates the activity of topoisomerase 2 and the Topoisomerase 3a–Rmi1–Rmi2 complex for the resolution of UFBs [12, 41]. Moreover, it has been shown that PICH ATPase activity is necessary for its DNA translocase activity and for UFB resolution [12]. Therefore, compounds blocking PICH ATPase activity are predicted to have antitumoral properties.

In the current study, we have specifically studied the role of PICH in BL, an aggressive non-Hodgkin B-cell lymphoma caused by translocations that lead to *C-MYC* overexpression. There are sporadic cases and endemic cases associated with HIV, Epstein-Barr Virus, and malaria. While most children respond well to standard chemotherapy (including cyclophosphamide, vincristine, prednisolone, and doxorubicin), many adults undergo a relapse of the disease that is associated with a poor prognosis. Moreover, the side effects associated with chemotherapy pose an important risk of infections and secondary pathologies, especially in developing countries. Thus, BL patients could benefit from a more specific and less toxic therapy. Although we have used BL as a cancer model, we believe that a therapy based on PICH inhibition could be useful for other cancer types with high PICH expression. However, higher PICH expression could just reflect changes in the cell cycle or the proliferation rate of some cancer types. Thus, the role of PICH should be investigated with experimental studies in each case, as was recently done for breast cancer [37]. Data from the DepMap portal indicate that PICH is essential in only 164 out of 1095 cancer cell lines (DepMap Public 23Q2+Score, Chronos). Contrary to our in vivo and in vitro data, BL cell lines are not significantly affected by PICH depletion according to DepMap portal data. One possible explanation for this discrepancy is that PICH was not efficiently knocked out in these screens since this was not verified by western blotting or other means.

It would be relevant to investigate mutations or other conditions that increase sensitivity to PICH deficiency, as well as mutations that could confer resistance to PICH inhibitors. In this regard, *MYC* amplification and MYC overexpression do not drive *ERCC6L* dependence in a pan-cancer analysis of data from the DepMap portal (DepMap Public 23Q2+Score, Chronos). These data reinforce the idea that PICH inhibition could be useful for other non-*MYC*-driven cancer types. Interestingly, a recent study has shown that PICH becomes more important for cell viability in cells deficient for DNA maintenance genes, such as those related to DNA repair, DNA condensation, centromere stability, and regulators of the replication fork stability such as FIRRM [42]. In addition, a recent study has found that PICH deficiency is synthetic lethal with SUMOylation inhibitors [43]. In this line, treatments combining PICH inhibitors and compounds targetting these DNA repair pathways could potentially offer higher efficacy.

In summary, the cumulative evidence in the literature of the relevance of PICH for the proliferation of different cancer cells, together with our results presented here using a *Pich* conditional KO model, indicate that PICH is a promising therapeutic target to treat BL and possibly other cancers with high PICH expression.

### Supplementary information

Supplementary figures

## Data Availability

The data that support the findings of this study are available on request to the corresponding author. andres.lopez@cabimer.es.
