# Supplementary material for: PICH deficiency limits the progression of MYC-induced B-cell lymphoma
Source: Blood Cancer J. 2024 Jan 23;14(1):16. doi: 10.1038/s41408-024-00979-y (PMC10803365; doi:10.1038/s41408-024-00979-y)
Supplement: Supplementary file 1 — Supplementary figures [file 41408_2024_979_MOESM1_ESM.pptx]

## Slide 1
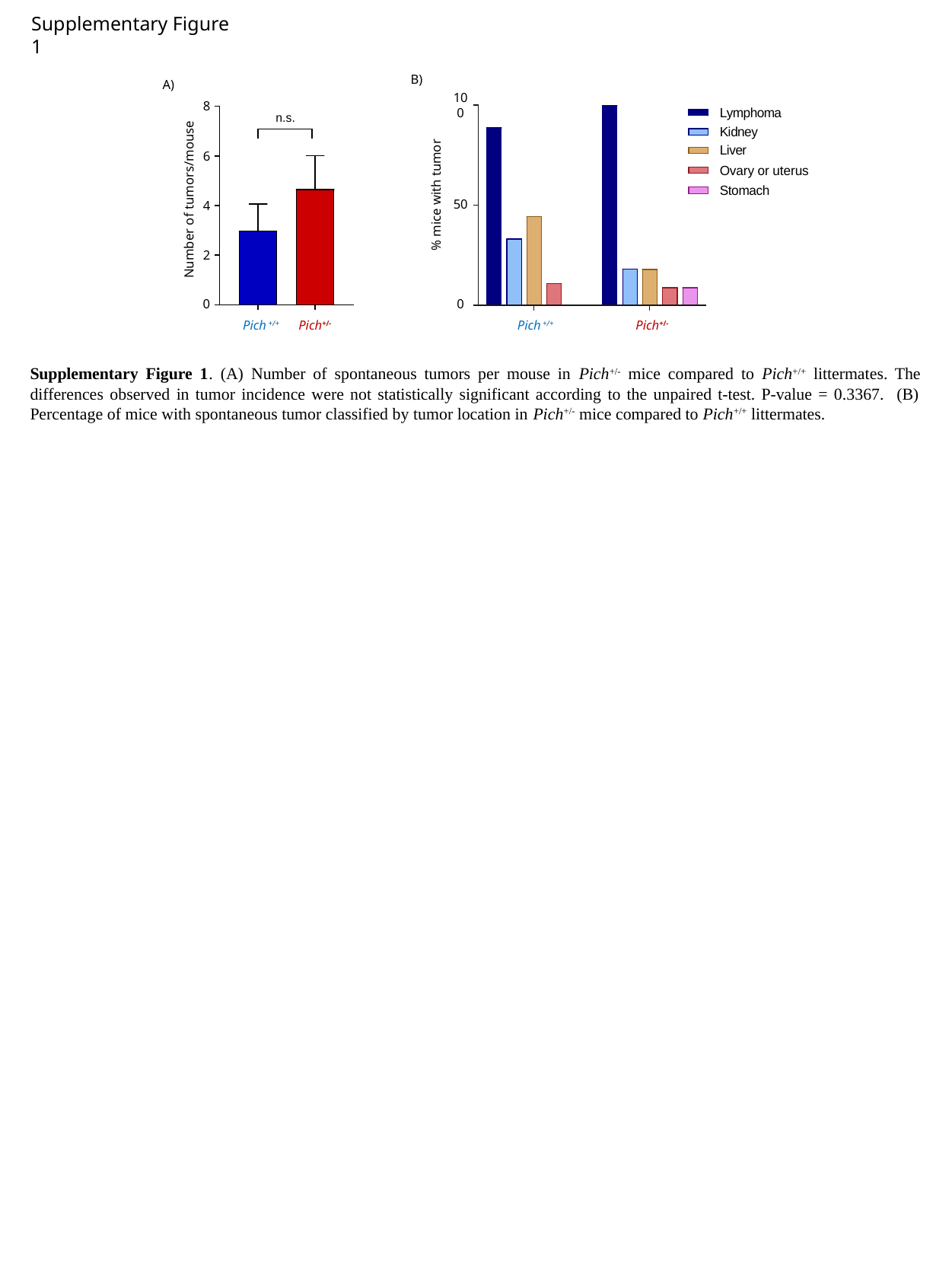

Supplementary Figure 1
B)
A)
8
Number of tumors/mouse
6
4
2
0
Pich +/+
Pich+/-
100
50
0
% mice with tumor
Pich +/+
Pich+/-
Supplementary Figure 1. (A) Number of spontaneous tumors per mouse in Pich+/- mice compared to Pich+/+ littermates. The differences observed in tumor incidence were not statistically significant according to the unpaired t-test. P-value = 0.3367. (B) Percentage of mice with spontaneous tumor classified by tumor location in Pich+/- mice compared to Pich+/+ littermates.

## Slide 2
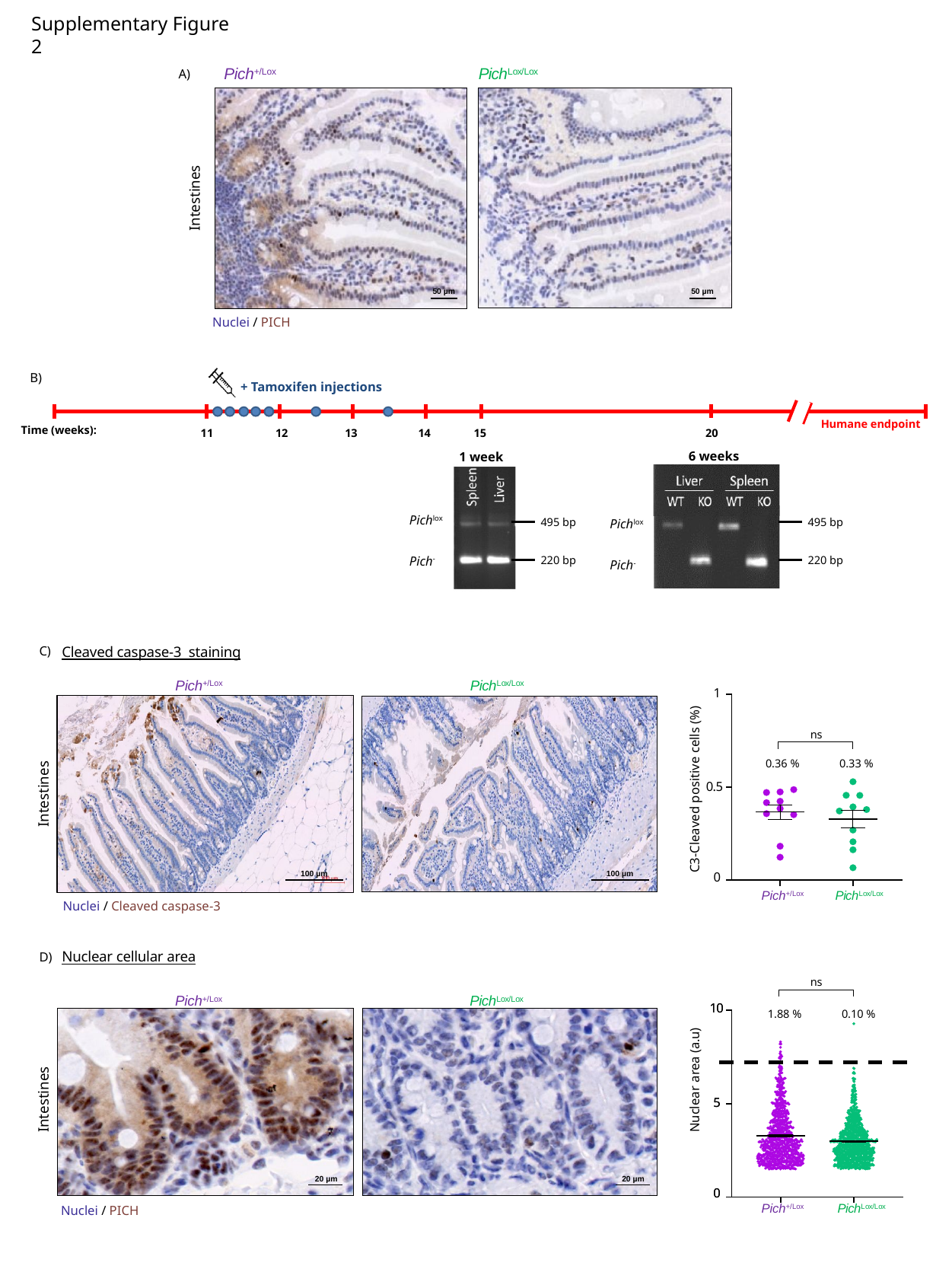

PichLox/Lox
Pich+/Lox
Supplementary Figure 2
A)
Intestines
50 µm
50 µm
Nuclei / PICH
B)
+ Tamoxifen injections
Humane endpoint
Time (weeks):
20
11
12
13
14
15
6 weeks
1 week
Pichlox
495 bp
495 bp
Pichlox
Pich-
220 bp
220 bp
Pich-
Cleaved caspase-3 staining
C)
Pich+/Lox
PichLox/Lox
1
0.5
0
ns
0.36 %
0.33 %
C3-Cleaved positive cells (%)
Intestines
100 µm
100 µm
Pich+/Lox
PichLox/Lox
Nuclei / Cleaved caspase-3
Nuclear cellular area
D)
ns
10
5
0
1.88 %
0.10 %
Nuclear area (a.u)
Pich+/Lox
PichLox/Lox
Pich+/Lox
PichLox/Lox
10
5
0
Intestines
20 µm
20 µm
Nuclei / PICH

## Slide 3
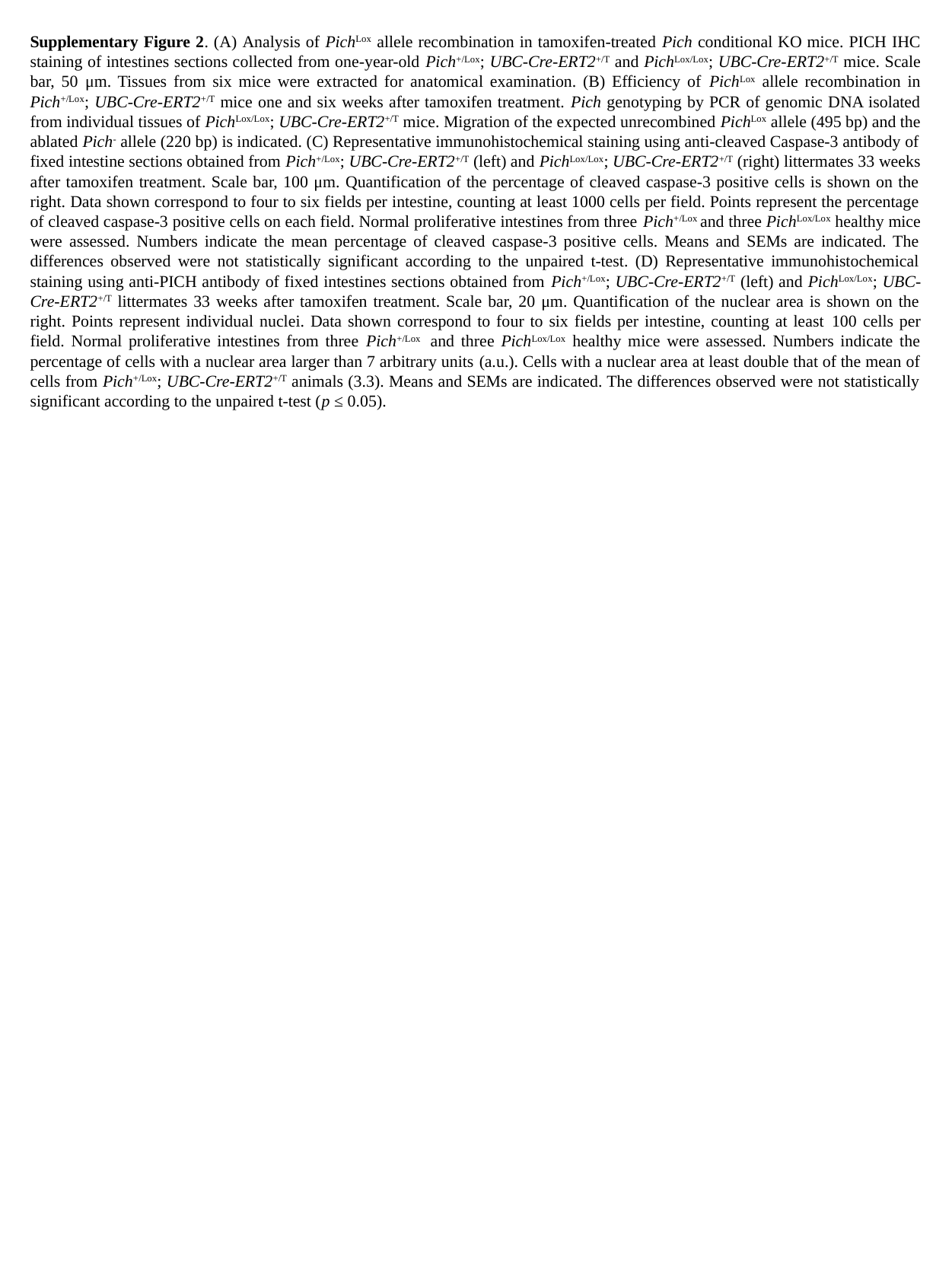

Supplementary Figure 2. (A) Analysis of PichLox allele recombination in tamoxifen-treated Pich conditional KO mice. PICH IHC staining of intestines sections collected from one-year-old Pich+/Lox; UBC-Cre-ERT2+/T and PichLox/Lox; UBC-Cre-ERT2+/T mice. Scale bar, 50 μm. Tissues from six mice were extracted for anatomical examination. (B) Efficiency of PichLox allele recombination in Pich+/Lox; UBC-Cre-ERT2+/T mice one and six weeks after tamoxifen treatment. Pich genotyping by PCR of genomic DNA isolated from individual tissues of PichLox/Lox; UBC-Cre-ERT2+/T mice. Migration of the expected unrecombined PichLox allele (495 bp) and the ablated Pich- allele (220 bp) is indicated. (C) Representative immunohistochemical staining using anti-cleaved Caspase-3 antibody of fixed intestine sections obtained from Pich+/Lox; UBC-Cre-ERT2+/T (left) and PichLox/Lox; UBC-Cre-ERT2+/T (right) littermates 33 weeks after tamoxifen treatment. Scale bar, 100 μm. Quantification of the percentage of cleaved caspase-3 positive cells is shown on the right. Data shown correspond to four to six fields per intestine, counting at least 1000 cells per field. Points represent the percentage of cleaved caspase-3 positive cells on each field. Normal proliferative intestines from three Pich+/Lox and three PichLox/Lox healthy mice were assessed. Numbers indicate the mean percentage of cleaved caspase-3 positive cells. Means and SEMs are indicated. The differences observed were not statistically significant according to the unpaired t-test. (D) Representative immunohistochemical staining using anti-PICH antibody of fixed intestines sections obtained from Pich+/Lox; UBC-Cre-ERT2+/T (left) and PichLox/Lox; UBC-Cre-ERT2+/T littermates 33 weeks after tamoxifen treatment. Scale bar, 20 μm. Quantification of the nuclear area is shown on the right. Points represent individual nuclei. Data shown correspond to four to six fields per intestine, counting at least 100 cells per field. Normal proliferative intestines from three Pich+/Lox and three PichLox/Lox healthy mice were assessed. Numbers indicate the percentage of cells with a nuclear area larger than 7 arbitrary units (a.u.). Cells with a nuclear area at least double that of the mean of cells from Pich+/Lox; UBC-Cre-ERT2+/T animals (3.3). Means and SEMs are indicated. The differences observed were not statistically significant according to the unpaired t-test (p ≤ 0.05).

## Slide 4
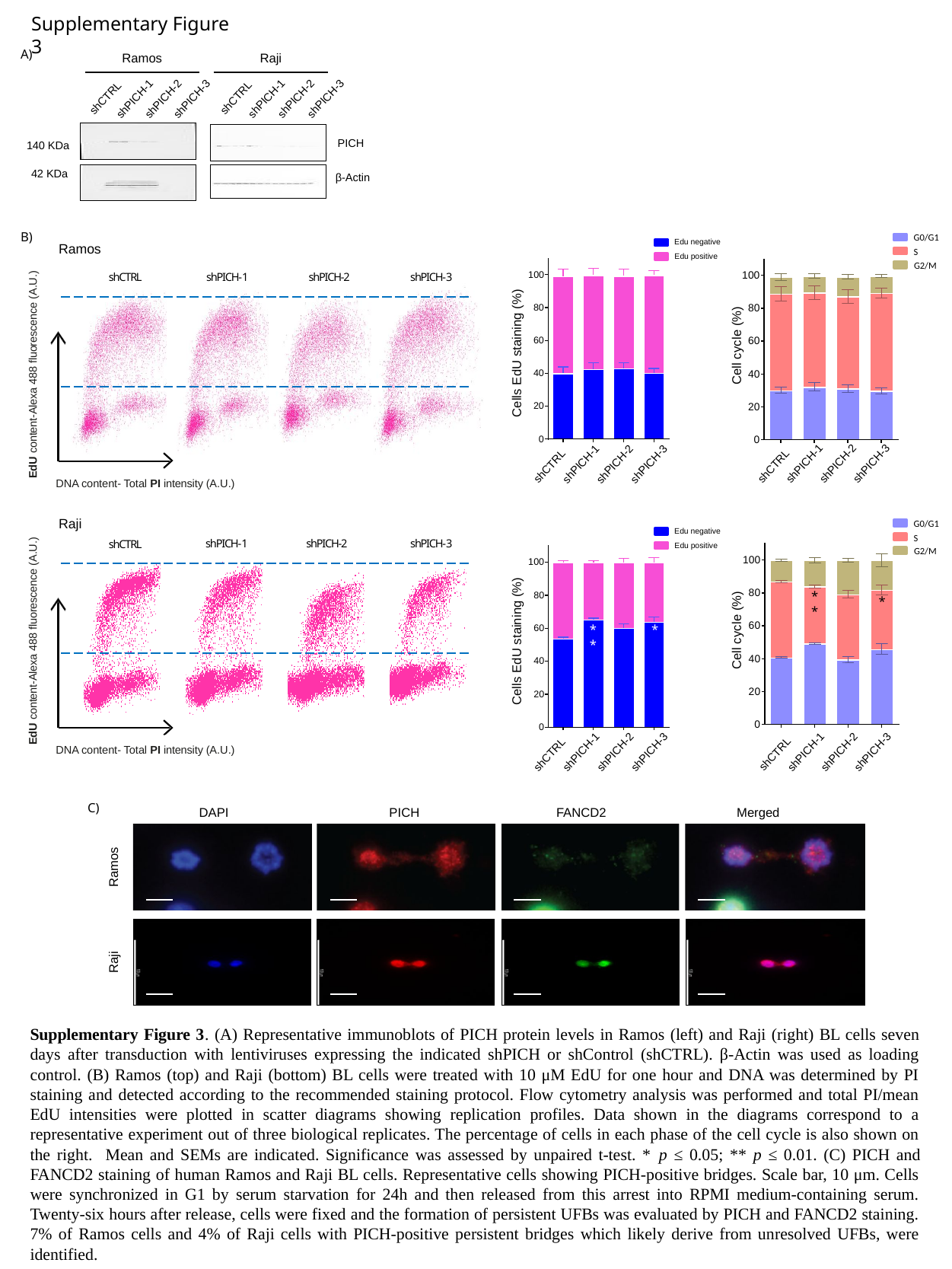

Supplementary Figure 3
A)
Ramos
Raji
shCTRL
shPICH-1
shPICH-2
shPICH-3
shCTRL
shPICH-1
shPICH-2
shPICH-3
PICH
140 KDa
42 KDa
β-Actin
G0/G1
S
G2/M
B)
Edu negative
Edu positive
Ramos
 EdU content-Alexa 488 fluorescence (A.U.)
shCTRL
shPICH-1
shPICH-2
shPICH-3
Cell cycle (%)
Cells EdU staining (%)
shPICH-1
shPICH-2
shPICH-3
shCTRL
shPICH-1
shPICH-2
shPICH-3
shCTRL
DNA content- Total PI intensity (A.U.)
 EdU content-Alexa 488 fluorescence (A.U.)
Raji
G0/G1
S
G2/M
Edu negative
Edu positive
shCTRL
shPICH-1
shPICH-2
shPICH-3
**
*
Cell cycle (%)
**
*
Cells EdU staining (%)
shPICH-1
shPICH-2
shPICH-3
shCTRL
shPICH-1
shPICH-2
shPICH-3
shCTRL
DNA content- Total PI intensity (A.U.)
C)
DAPI
PICH
FANCD2
Merged
Ramos
Raji
Supplementary Figure 3. (A) Representative immunoblots of PICH protein levels in Ramos (left) and Raji (right) BL cells seven days after transduction with lentiviruses expressing the indicated shPICH or shControl (shCTRL). β-Actin was used as loading control. (B) Ramos (top) and Raji (bottom) BL cells were treated with 10 μM EdU for one hour and DNA was determined by PI staining and detected according to the recommended staining protocol. Flow cytometry analysis was performed and total PI/mean EdU intensities were plotted in scatter diagrams showing replication profiles. Data shown in the diagrams correspond to a representative experiment out of three biological replicates. The percentage of cells in each phase of the cell cycle is also shown on the right. Mean and SEMs are indicated. Significance was assessed by unpaired t-test. * p ≤ 0.05; ** p ≤ 0.01. (C) PICH and FANCD2 staining of human Ramos and Raji BL cells. Representative cells showing PICH-positive bridges. Scale bar, 10 μm. Cells were synchronized in G1 by serum starvation for 24h and then released from this arrest into RPMI medium-containing serum. Twenty-six hours after release, cells were fixed and the formation of persistent UFBs was evaluated by PICH and FANCD2 staining. 7% of Ramos cells and 4% of Raji cells with PICH-positive persistent bridges which likely derive from unresolved UFBs, were identified.

## Slide 5
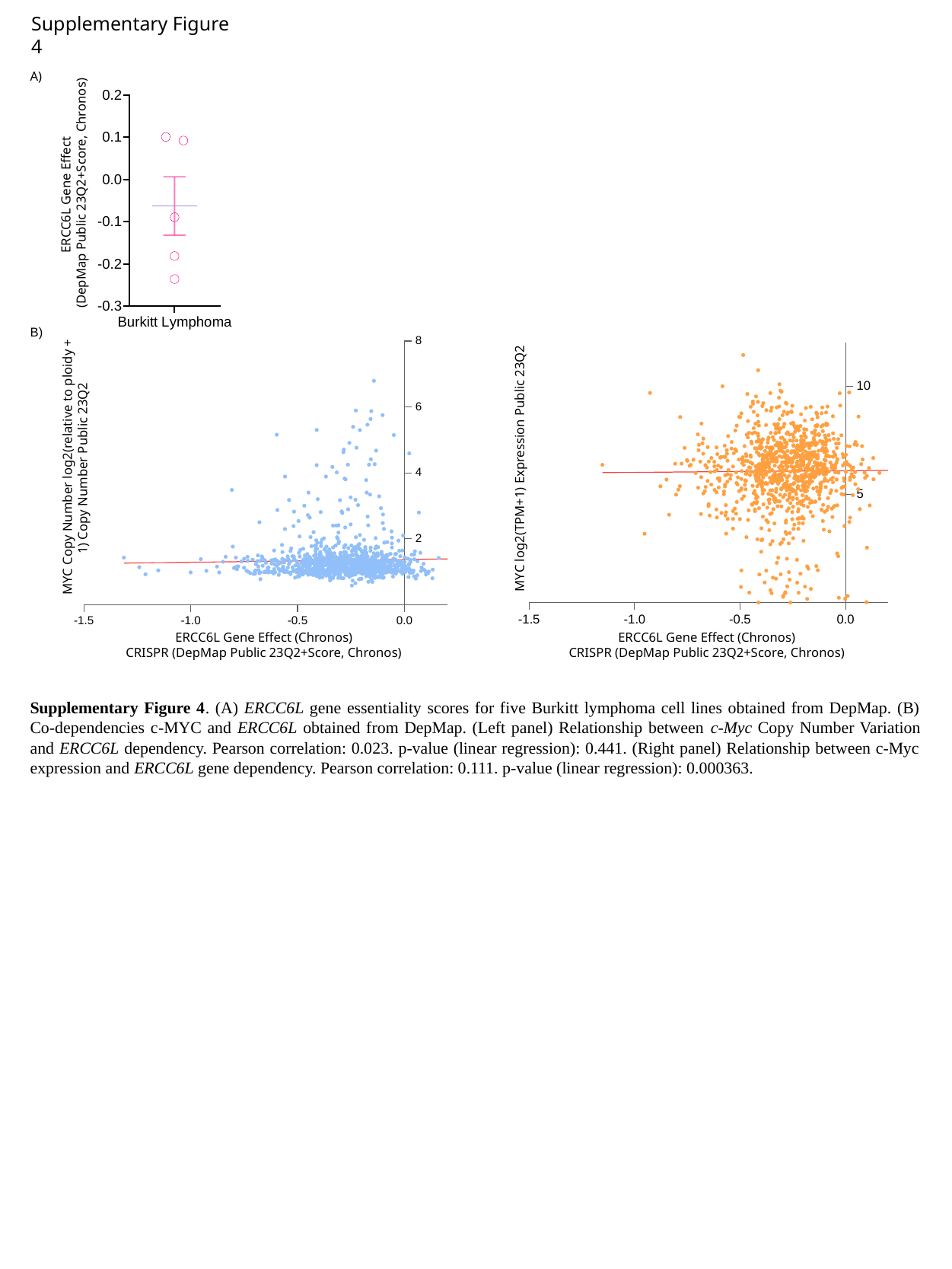

Supplementary Figure 4
A)
ERCC6L Gene Effect
(DepMap Public 23Q2+Score, Chronos)
B)
MYC Copy Number log2(relative to ploidy + 1) Copy Number Public 23Q2
MYC log2(TPM+1) Expression Public 23Q2
ERCC6L Gene Effect (Chronos)
CRISPR (DepMap Public 23Q2+Score, Chronos)
ERCC6L Gene Effect (Chronos)
CRISPR (DepMap Public 23Q2+Score, Chronos)
Supplementary Figure 4. (A) ERCC6L gene essentiality scores for five Burkitt lymphoma cell lines obtained from DepMap. (B) Co-dependencies c-MYC and ERCC6L obtained from DepMap. (Left panel) Relationship between c-Myc Copy Number Variation and ERCC6L dependency. Pearson correlation: 0.023. p-value (linear regression): 0.441. (Right panel) Relationship between c-Myc expression and ERCC6L gene dependency. Pearson correlation: 0.111. p-value (linear regression): 0.000363.
